# Mapping the Effect of Gly Mutations in Collagen on α2β1 Integrin Binding*

**DOI:** 10.1074/jbc.M116.726182

**Published:** 2016-07-18

**Authors:** Sezin Yigit, Hongtao Yu, Bo An, Samir Hamaia, Richard W. Farndale, David L. Kaplan, Yu-Shan Lin, Barbara Brodsky

**Affiliations:** From the Departments of ‡ Biomedical Engineering and; § Chemistry, Tufts University, Medford, Massachusetts 02155 and; ¶ Department of Biochemistry, University of Cambridge, Cambridge CB2 1QW, United Kingdom

**Keywords:** collagen, extracellular matrix, integrin, molecular dynamics, recombinant protein expression, osteogenesis imperfecta, binding, missense mutations, triple helix

## Abstract

The replacement of one Gly in the essential repeating tripeptide sequence of the type I collagen triple helix results in the dominant hereditary bone disorder osteogenesis imperfecta. The mechanism leading to pathology likely involves misfolding and autophagy, although it has been hypothesized that some mutations interfere with known collagen interactions. Here, the effect of Gly replacements within and nearby the integrin binding GFPGER sequence was investigated using a recombinant bacterial collagen system. When a six-triplet human type I collagen sequence containing GFPGER was introduced into a bacterial collagen-like protein, this chimeric protein bound to integrin. Constructs with Gly to Ser substitutions within and nearby the inserted human sequence still formed a trypsin-resistant triple helix, suggesting a small local conformational perturbation. Gly to Ser mutations within the two Gly residues in the essential GFPGER sequence prevented integrin binding and cell attachment as predicted from molecular dynamics studies of the complex. Replacement of Gly residues C-terminal to GFPGER did not affect integrin binding. In contrast, Gly replacements N-terminal to the GFPGER sequence, up to four triplets away, decreased integrin binding and cell adhesion. This pattern suggests either an involvement of the triplets N-terminal to GFPGER in initial binding or a propagation of the perturbation of the triple helix C-terminal to a mutation site. The asymmetry in biological consequences relative to the mutation site may relate to the observed pattern of osteogenesis imperfecta mutations near the integrin binding site.

## Introduction

The extracellular matrix protein collagen is the most abundant protein in humans. The defining feature of collagens is the triple helix molecular structure composed of three supercoiled polyproline II helices (1, 2). Animal collagens are also unique in their high content of hydroxyproline (Hyp)^4^ important for triple helix stability. The most common collagen is type I collagen, which forms banded fibrils with a 67-nm axial periodicity in bone, tendon, skin, cornea, and other tissues. Type I collagen is a heterotrimeric triple helix consisting of two α1(I) chains and one α2(I) chain coded by the *COL1A1* and *COL1A2* genes. The triple helical conformation requires the smallest amino acid, Gly, as every third residue to stabilize the tightly packed structure, generating the characteristic collagen (Gly-Xaa-Yaa)*_n_* sequence (3, 4). The essential nature of Gly is shown by the pathology that results when even one Gly is replaced by a larger residue. Mutations in either of the genes for type I collagen are known to lead to the dominant form of the fragile bone disease osteogenesis imperfecta (OI) (5–7) with a highly variable phenotype ranging from mild to perinatal lethal. The most common kind of mutations observed for OI cases are single base changes that lead to the replacement of one Gly in the (Gly-Xaa-Yaa)*_n_* repeating sequence of the triple helix by another amino acid residue, and such mutations have been reported at almost two-thirds of the Gly locations along the triple helix. Single base replacements in Gly codons can lead to eight different amino acids (Ser, Ala, Cys, Arg, Asp, Glu, Val, and Trp), and Ser is the most frequently observed replacement residue in OI. The pathway leading from a Gly replacement in collagen to bone fragility is under active investigation.

Different approaches have been applied to elucidate the consequences of a Gly substitution in type I collagen. There is structural evidence from x-ray crystallography, NMR, and physicochemical studies on collagen model peptides that replacement of one Gly in the repeating tripeptide sequence by a larger residue can distort the triple helix structure near the mutation site, disrupt interchain hydrogen bonding, produce local destabilization, and cause a dislocation in the superhelix register (8–10). Studies on collagens produced by OI fibroblasts indicate that a Gly substitution slows down triple helix folding, and such a folding delay leads to excess post-translational modification because these enzymatic modifications can only act on unfolded chains (11, 12). Studies on OI mouse models have shown abnormal procollagen retention in the endoplasmic reticulum, increased intracellular breakdown via degradative pathways, and osteoblast malfunction (13–15). Although abnormal collagens may be degraded intracellularly, some mutant collagens are secreted and incorporated into fibrils (13, 16, 17). There is a large database of OI mutations for *COL1A1* and *COL2A1* (5, 6), and analysis of known mutation sites has led to the suggestion that some mutations may interfere with biological interactions involving collagen and that interaction mutations could be particularly severe or even non-viable (7, 18).

Type I collagen interacts with many matrix and cell receptor proteins, including the integrin cell receptors (α2β1, α1β1, α10β1, and α11β1) (19). Only the native triple helical collagen will bind integrins, which do not interact with denatured collagen. The precise (Gly-Xaa-Yaa)*_n_* amino acid sequence in collagen required for integrin binding has been determined through the use of collagen fragments and triple helical peptides (20). Peptide studies showed that the two triplets GFOGER are necessary and sufficient for collagen binding to integrins (21), and GFOGER constitutes the strongest binding sequence in collagens, although other weaker sites are also recognized (20). The sequence GFPGER without Hyp (O) also binds integrins but with lower affinity (21, 22). The high resolution crystal structure of a co-crystal of the I domain of the α2 subunit of integrin bound to the triple helical peptide (GPO)_2_GFOGER(GPO)_3_ at 2.1-Å resolution has been reported (23). The integrin I domain shows many interactions with the GFOGER sequence of the middle strand and the trailing strand of the triple helix, including coordination of the metal ion in the MIDAS motif with the Glu in the GFOGER sequence. Because the binding of the collagen triple helix to α2β1 integrin is so well defined, this system was chosen to investigate the effect of Gly missense mutations on a known biological interaction.

A bacterial recombinant system was used to examine the effect of mutations on collagen-integrin interactions. The protein VCL is based on a collagen-like Scl2 protein found in *Streptococcus pyogenes* with an N-terminal trimerization domain denoted as V and an uninterrupted triple helix collagen-like (CL) domain, (Gly-Xaa-Yaa)_79_ (24, 25). The VCL protein expressed in *Escherichia coli* has a thermal stability near 37 °C, a stability achieved in the absence of Hyp because *E. coli* lacks the post-translational enzymes necessary for hydroxylation of Pro (26). VCL does not have any intrinsic biological activity and has proved to be a successful platform for insertion of specific human collagen binding sequences, including GFPGER and other integrin binding motifs (22, 27–31). Six Gly-Xaa-Yaa triplets from the human α1(I) chain residues 496–513, which include GFPGER and two surrounding triplets on each side, were inserted within the bacterial CL triple helix domain. This chimeric construct was shown to bind to integrin and promote cell adhesion. Constructs were then generated with a Gly to Ser mutation at each Gly site within the insertion. As expected, a mutation at either of the two Gly residues within the GFPGER sequence disrupted binding to the integrin α2 I domain and diminished cell adhesion. Surprisingly, mutations in the four triplets N-terminal to the GFPGER sequence also decreased integrin binding and cell adhesion, whereas mutations C-terminal to GFPGER had no effect. This asymmetric pattern can be explained in part by molecular dynamics simulations, which showed that a Gly to Ser replacement preferentially disrupts the triple helix C-terminal to the mutation site.

## Results

### 

#### 

##### Generation and Structural Characterization of Recombinant Collagen Proteins with Gly to Ser Mutations in the Integrin Binding Sequence

**FIGURE 1.**
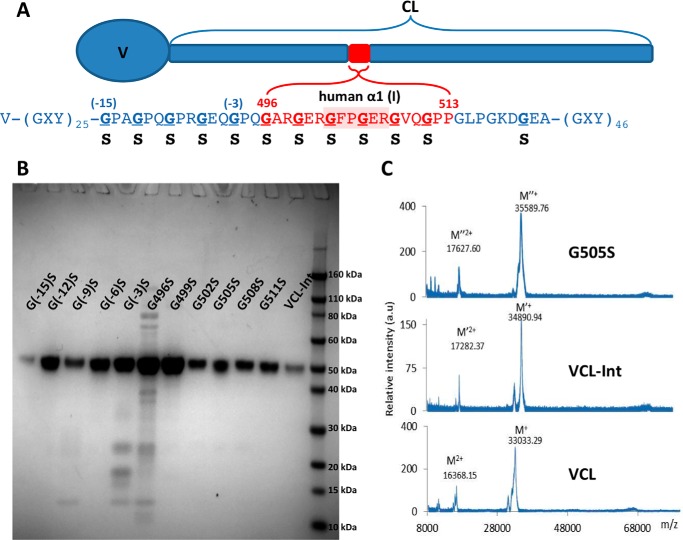
*A*, schematic diagram of recombinant bacterial collagen VCL-Int with the insertion of human α1(I) chain residues from Gly-496 to Pro-513 (*red*), including the integrin α2β1 binding sequence, GFPGER. Residue numbers are taken from the UniProt entry P02452, and numbering starts from the beginning of the triple helix region. Bacterial collagen residues (*blue*) are denoted by their position relative to the human sequence insertion G(−12)POG(−9)PRG(−6)EQG(−3)PQGARGERGFPGERGVQGPP. *Underlined* Gly residues are mutated to Ser individually. *B*, SDS-polyacrylamide gel showing all the recombinant proteins, including VCL-Int and the proteins with Gly replacements. The *right lane* contains the Novex Sharp protein standard (Invitrogen); collagen monomer chains run slower than expected as reported previously. *C*, MALDI-TOF spectrometry of VCL, VCL-Int, and a representative mutated protein (G505S). *a.u.*, arbitrary units.

**FIGURE 2.**
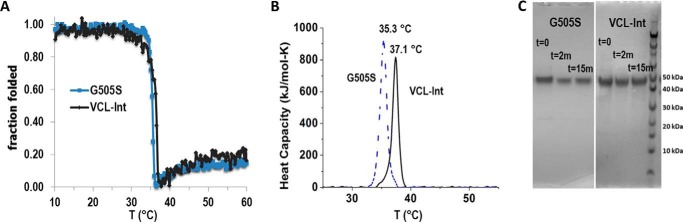
**Structural characterization of VCL-Int and a representative mutated construct (G505S).** *A*, CD thermal transitions of VCL-Int and G505S showing triple helix unfolding around 37 °C. The increase in ellipticity after 37 °C is due to the unfolding of the α-helix in the V domain. *B*, differential scanning calorimetry analysis showing thermal transitions of 37.1 °C for VCL-Int and 35.3 °C for G505S. *C*, SDS-PAGE of VCL-Int and VCL-Int G505S after trypsin digestion for time *t* = 0, 2, and 15 min at 20 °C. Similar results were observed for G496S, G499S, G502S, G508S, and G511S (data not shown). A small initial drop in intensity is observed that could reflect rapid digestion of a small amount of impurity or unfolded collagen. There is little change in the intensity following this initial decrease, suggesting that the mutations did not lead to major unfolding.

A recombinant bacterial collagen-like protein, designated VCL-Int, was generated with an insertion of six triplets from human α1(I) chain residues 496–513, GARGERGFPGERGVQGPP, into a central part of the *S. pyogenes* Scl2 (Gly-Xaa-Yaa)_79_ CL domain (Fig. 1*A*). Single base point mutations were then introduced to convert each Gly codon to Ser within and surrounding the (Gly-Xaa-Yaa)_6_ insertion (Fig. 1*A*). All constructs showed one strong band that corresponds to the collagen monomer on SDS-PAGE, and the molecular masses (∼34 kDa) were confirmed by MALDI-TOF (Fig. 1, *B* and *C*). The VCL-Int protein has the same thermal stability as VCL, and the introduction of Gly to Ser mutations at different sites resulted in a small decrease in thermal stability (1–2 °C) in circular dichroism (CD) melting curves and differential scanning calorimetry (Fig. 2, *A* and *B*). Trypsin treatment of VCL-Int and the VCL-Int proteins with Gly replacements led to a small initial decrease in intensity of the original SDS-PAGE band to a level that was then maintained (Fig. 2*C*). Because the tightly packed triple helix is resistant to trypsin digestion, these results indicate that the mutations did not cause a major structural perturbation. It is likely that the small amount of trypsin-susceptible material represented some unfolded collagen or minor impurity that could not be fully separated from the triple helical collagen.

##### Effect of Gly to Ser Mutations on Integrin Binding

**FIGURE 3.**
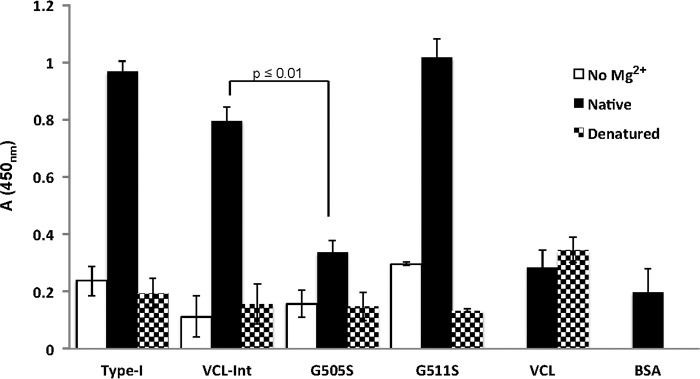
**Solid-state analysis of recombinant integrin α2 I domain binding to native and denatured recombinant collagens that are immobilized on 96-well plates and incubated with recombinant I domain (10 μg/ml).** Anti-GST antibody was used to detect the bound I domain. Proteins were immobilized in their native state (*black columns*), in the denatured state (*dotted columns*), and in the absence of the Mg^2+^ cation (*white columns*). The native VCL showed no binding, whereas native VCL-Int showed a high binding similar to native type I collagen. Native G505S had a low binding, whereas native G511S had a binding similar to VCL-Int. All proteins in their denatured states showed no binding as triple helicity is essential for integrin binding. The divalent cation (Mg^2+^) is essential for the MIDAS motif, and BSA was used as a background value. Statistical analysis was performed by paired *t* test. The binding of G505S was significantly different from the binding of VCL-Int (*p* ≤ 0.01). All experiments were in triplicate, and *error bars* represent the standard deviation.

Solid-state binding assays were utilized to determine the binding of integrin to recombinant proteins with Gly to Ser replacements. Recombinant I domain from the integrin α2 subunit was used for the binding assays as described previously (21). The control VCL protein did not show any binding to the integrin I domain, establishing it as a negative control that does not interact with the I domain (24). VCL-Int, with the GARGERGFPGERGVQGPP insertion, showed strong, specific binding to the I domain (Fig. 3). Denatured constructs did not bind to integrin, confirming the requirement for the triple helix structure. Omission of the divalent cation (Mg^2+^), required for the MIDAS motif, also eliminated activity (Fig. 3).

**FIGURE 4.**
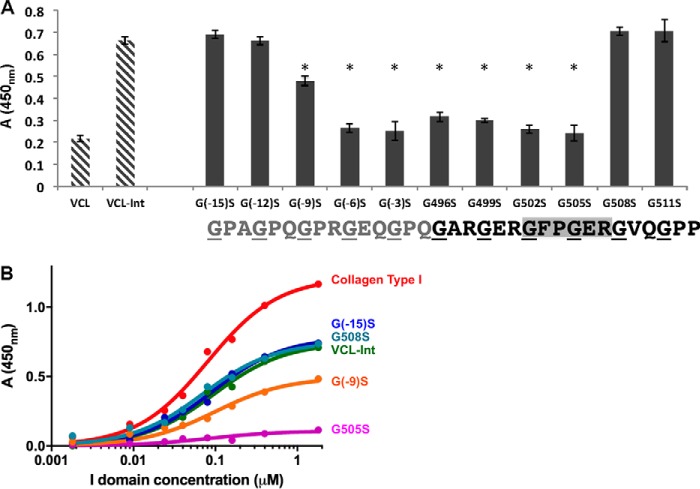
*A*, solid-state analysis of recombinant α2 I domain binding to all recombinant bacterial collagens containing Gly to Ser substitutions at an I domain concentration of 0. 4 μm. The two *striped columns* on the *left* are the negative control VCL with no binding and the wild-type VCL-Int with strong binding. Experiments were carried out in triplicate, and *error bars* represent the standard deviation. Statistical analysis was performed with the paired *t* test. Columns marked with an *asterisk* showed a statistically significant difference in binding compared with the VCL-Int control (*p* ≤ 0.01). *B*, dose response of I domain binding to recombinant collagens adsorbed onto 96-well plates. Bound protein was detected with antibodies and measured as absorbance at 450 nm. *Red*, collagen type I; *green*, VCL-Int; *cyan*, G508S; *purple*, G505S; *orange*, G(−9)S; *blue*, G(−15)S. Data were fit in GraphPad Prism® using a non-linear fit (one site, specific binding). Error bars are not included because only one set of experiments can be carried out on a given 96-well plate, and it is not possible to average values between different plates due to slight changes in conditions. All assays have been independently repeated at least three times on new plates to confirm that results are consistent.

Gly to Ser replacements at either of the two Gly residues within the essential GFPGER binding site, G502S and G505S, abolished integrin binding, showing values similar to the BSA negative control (Fig. 4*A*). To define how far the binding footprint extends from the essential two triplets, experiments were performed on constructs with mutations both C-terminal and N-terminal to GFPGER. In a C-terminal direction, Gly to Ser mutations G508S and G511S did not affect integrin binding (Fig. 4*A*). A more distant Gly to Ser mutation within the bacterial sequence, three triplets C-terminal to the insert, was also generated as a control and showed the same high binding as the VCL-Int (data not shown).

In contrast, mutations at the two Gly sites N-terminal to the GFPGER sequence, G499S and G496S, showed a dramatic decrease in binding (Fig. 4*A*). To establish the boundary for the recovery of integrin binding, constructs with Gly to Ser mutations at sites N-terminal to the human sequence insertion were generated within the bacterial collagen sequence. The notation for these replacements is relative to the insertion sequence, *e.g.* G(−3)S for the Gly three residues N-terminal to the insertion sequence residue Gly496. Both G(−3)S and G(−6)S constructs had very low binding, whereas G(−9)S showed intermediate binding, higher than G(−6)S but lower than VCL-Int. Constructs with mutations still further N-terminal to the insert, G(−12)S and G(−15)S, exhibited strong integrin binding similar to that of the control VCL-Int (Fig. 4*A*).

The binding assays described above were carried out at an I domain concentration of 0.2 μm.^5^ A range of I domain concentrations from 0 to 1.8 μm was used to obtain titration curves for a representative set of the recombinant constructs: the VCL-Int control, a mutation within the GFPGER binding sequence (G505S), one mutation C-terminal to that binding sequence (G508S), a mutation five triplets N-terminal to the binding site G(−9)S, and a mutation seven triplets N-terminal to the binding site G(−15)S (Fig. 4*B*). These titration curves confirm the trend seen for binding in Fig. 4*A*, but meaningful binding constants cannot be calculated from such curves due to the non-linear effects inherent in solid-state binding assays (32).

##### Effect of Gly to Ser Mutations on Cell Adhesion of HT1080 Cells

**FIGURE 5.**
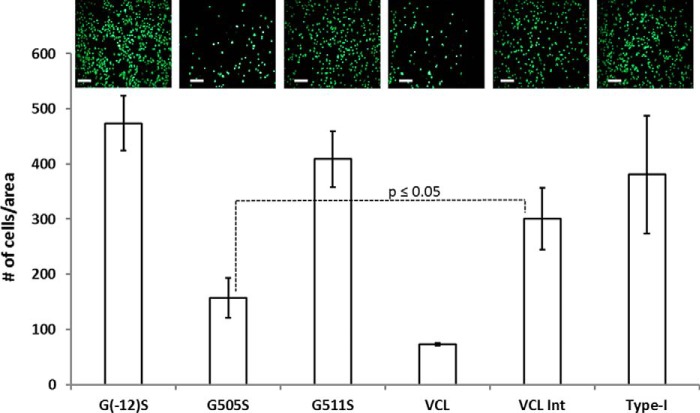
**HT1080 cell adhesion assay shows the *in vitro* binding profile of recombinant proteins with and without mutations.** Live imaging (shown on *top*) was performed after calcein-AM staining of bound cells. *Scale bars*, 50 μm. Quantitative analysis for the cell attachment was performed by ImageJ. Experiments were carried out in triplicate with *error bars* representing the standard deviation and statistical analysis performed with the paired *t* test. G505S had a statistically significant decrease in cell adhesion compared with VCL-Int (*p* ≤ 0.05).

To test binding affinities in a biological context, representative constructs were examined for their adhesion to HT1080 cells, which are known to express integrin α2β1 as the only collagen-binding integrin receptor. Cell adhesion profiles were observed by cell staining followed by live imaging and cell count comparison. HT1080 cells showed very low adhesion to VCL but showed a strong binding to wells coated with type I collagen or VCL-Int. The G505S construct that has the Gly to Ser mutation within the GFPGER binding region showed a very low cell adhesion similar to the VCL construct, confirming the low binding. G511S, chosen as representative of mutations C-terminal to the binding region, showed a high cell adhesion profile similar to VCL-Int. The G(−12)S construct represents the recovery of binding if one goes sufficiently far in an N-terminal direction from the integrin binding site, and it showed cell adhesion similar to that of VCL-Int (Fig. 5). To confirm the cell adhesion results, crystal violet staining was also performed with both native and denatured G(−12)S, G505S, VCL-Int, VCL, and type I collagen. The staining experiments showed the same decrease in cell adhesion for G505S as seen by live imaging (data not shown). Thus, *in vitro* cell adhesion studies correlate well with the solid-state binding assay results.

##### Molecular Dynamics Simulations for a Triple Helix with Gly to Ser Replacements within or nearby the Integrin Binding Sequence

**FIGURE 6.**
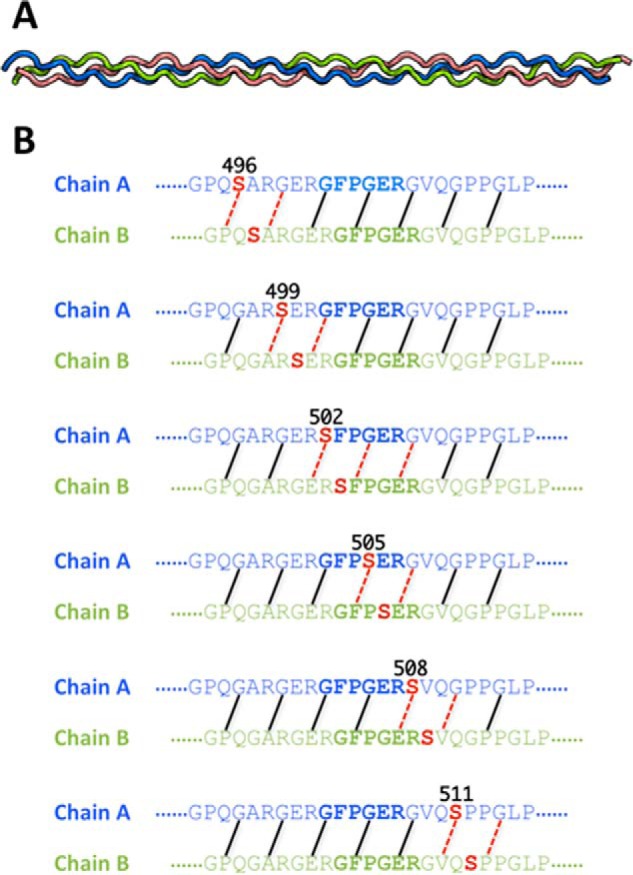
*A*, the triple helix structure of the wild-type collagen peptide generated with the Triple-Helical Collagen Building Script (THeBuScr) (47). Chains A, B, and C are colored in *blue*, *green*, and *red*, respectively. *B*, interchain backbone NH···CO interactions are illustrated. Different backbone NH···CO interactions are disrupted in the mutants depending on the location of the substitution. Backbone hydrogen bonds whose average N···O distances were ≥3.5 Å during the course of simulation are labeled with *red dashed lines* to indicate their disruption. The hydrogen bonding is shown only for chains A and B of the triple helix because these are the chains that interact with integrin (23).

Molecular dynamics (MD) simulations were performed to explore the conformational consequences of Gly to Ser replacements on the triple helix region containing the integrin binding sequence of human type I collagen surrounded by the bacterial collagen sequence. The simulations were carried out initially on a control triple helical peptide modeling VCL-Int that contained the six-triplet GARGERGFPGERGVQGPP human collagen insertion flanked by three triplets from the bacterial collagen sequence adjacent to the insert and finally capped by (GPO)_3_ stabilizing triplets on both ends (Fig. 6). MD simulations were also performed on a homologous sequence with a Gly to Ser replacement at positions G496S, G499S, G502S, G505S, G508S, and G511S. Interchain hydrogen bonds NH (Gly)···C=O (Xaa) are important for triple helix stability, so the distances were monitored throughout the triple helix. Each mutation led to a local disruption of hydrogen bonding at the mutation site where the NH (Ser) can no longer form a direct hydrogen bond with C=O in the adjacent chain. In addition, one to two hydrogen bonds are disrupted C-terminal to the mutation site. For instance, the mutation at position G499S (N-terminal to the integrin binding site) leads to the loss of the direct hydrogen bond at Ser-499 and perturbs hydrogen bonding within the GFP sequence C-terminal to it.

An analogous MD study was carried out for a triple helix peptide where the central human collagen α1(1) sequence GARGERGFPGERGVQGPP was surrounded by the actual sequence found in the human α1(1) chain rather than by the bacterial triplets (data not shown). The results were very similar, making it unlikely that the presence of the bacterial sequence, rather than the adjacent human α1(1) sequence, has any significant effect.

##### Molecular Dynamics Simulations on Integrin-Collagen Binding Complexes

**FIGURE 7.**
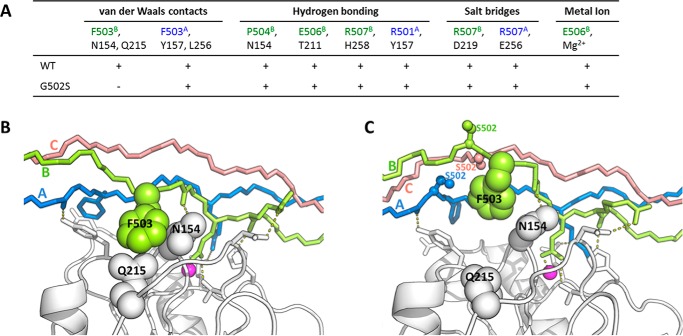
*A*, the key interactions that participate in the integrin-collagen binding and the residues involved in each interaction. The residues from integrin and chains A and B of collagen are colored in *black*, *green*, and *blue*, respectively. All these interactions are observed to be stable in the 100-ns MD simulation of integrin with WT collagen (indicated by + *symbols*). The van der Waals interaction of collagen Phe-503^B^ with integrin Gln-215 and Asn-154 is disrupted by the Gly to Ser mutations at position 502 (indicated by the − *symbol*). *B* and *C*, the simulation structures of integrin with WT collagen (*B*) and integrin with G502S mutant (*C*). Chains A, B, and C for collagens are colored in *blue*, *green*, and *red*, respectively. The Mg^2+^ ions are represented by *magenta spheres*. The substituted Ser residues are shown in *ball-and-stick*. The disrupted van der Waals interaction is illustrated.

To study how the Gly to Ser replacements influence the binding of collagen to integrin, MD simulations of integrin-collagen complexes, based on the crystal structure (23), were performed. Key interactions of the integrin-collagen binding were identified in the complex (Protein Data Bank code 1DZI) involving the middle chain B and the trailing chain A of the triple helix: 1) van der Waals contacts of collagen Phe-503^B^ side chain with side chains of integrin Asn-154 and Gln-215, 2) van der Waals contacts between collagen Phe-503^A^ and integrin Tyr-157 and Leu-256, 3) hydrogen bonding between collagen Hyp-504^B^ carbonyl group to integrin Asn-154, 4) hydrogen bond between the side chain of collagen Glu-506^B^ and the side chain of integrin Thr-211, 5) hydrogen bonding between collagen Arg-507^B^ carbonyl and integrin His-258 side chain, 6) hydrogen bonding between Arg-501^A^ carbonyl group in the triple helix and integrin Tyr-157, 7) hydrogen bonding between collagen Hyp-504^A^ side chain and integrin Asn-154 main chain, 8) salt bridges between collagen Arg-507^B^ and integrin Asp-219, 9) salt bridges between collagen Arg-507^A^ and integrin Glu-256, and 10) interaction between collagen Glu-506^B^ and metal ion. These interactions were monitored in our MD simulations (Fig. 7) except the interaction involving the hydroxyl group of Hyp (interaction 7 in the list), which is not included in the complexes because Hyp is absent in our recombinant bacterial collagen.

These interactions were monitored for MD simulations for Gly replacements in all six positions within the GARGERGFPGERGVQGPP human sequence. The simulations reveal that when the Gly at position 502 (**G**FPGER) is replaced by Ser MD simulations show the interaction of the F503^B^ side chain of collagen with the side chains Gln-215 and Asn-154 residues of integrin (interaction 1 above) was disrupted. It appears that the local perturbations in the triple helix near Ser-502 affect the relation between the collagen A and B chains and the integrin side chains (Fig. 7). When Gly at position 505 (GFP**G**ER) is replaced by Ser, the hydrogen bond between the backbone carboxyl group of collagen Arg-501^A^ and the hydroxyl group of integrin Tyr-157 (interaction 6 above) is lost (data not shown). These observations are consistent with the loss of binding for constructs with G502S and G505S mutations within the essential GFPGER sequence. When the Gly is replaced by Ser at position 508, the simulation shows that the important salt bridge between the collagen Arg-507 and Asp-219 of the integrin (interaction 8 above) is relatively stable despite small localized perturbations (data not shown). No disruptions of key interactions were seen for the mutation G511S. Mutations N-terminal to the GFPGER site at G499S and G496S led to a small perturbation in several interactions between integrin and collagen in some runs of the simulations, but the results were not definitive.

## Discussion

The interaction between the integrin α2β1 and collagen has been extensively studied because of its importance in cell adhesion and signaling. Several integrin binding sequences within collagen have been defined using triple helical peptides with GFOGER being the most effective (33–35). Recombinant collagens have also been used to characterize and manipulate integrin binding. Recombinant human type III collagen expressed in yeast promoted cell binding; this binding could be eliminated through removal of the five known integrin binding sites and restored through insertion of GFOGER (36, 37). The insertion of the human type I collagen cell binding sequences GFPGER or GLPGER within the collagen domain of the bacterial collagen protein Scl2 resulted in integrin binding and cell adhesion (22, 29, 30), and insertion of distinctive sequences could be used to form hydrogels with selective adhesion to endothelial cells *versus* smooth muscle cells (38). Peptides and recombinant collagen studies confirmed that integrin binding is dependent on the native triple helix conformation and showed that Hyp is not essential to binding (21, 22). Here, the introduction of human collagen α1(I) residues 496–513, GARGERGFPGERGVQGPP, into the *S. pyogenes* Scl2 collagen-like protein endowed the protein with integrin binding activity in a metal ion-dependent manner, and this recombinant system was used to examine the consequences of replacing Gly residues within and adjacent to the GFPGER integrin binding sequence.

The replacement of Gly by Ser at either of the residues Gly-502 and Gly-505 within the essential GFPGER sequence decreased integrin binding to baseline levels and prevented cell adhesion. Studies on model peptides have shown that a Gly to Ser replacement will provide a highly localized distortion and destabilization of the triple helix structure (8–10). Here, computational simulations of the triple helix alone showed a local disruption of interchain hydrogen bonding at each mutation site, and molecular dynamics simulations of the triple helix-I domain complex with Gly to Ser replacements at these two positions showed alterations in key interactions, consistent with the loss of binding activity. It is important to note that in the presence of these local conformational perturbations the tightly packed triple helix structure is maintained because the mutant collagens retain resistance to trypsin and show little change in global thermal stability.

Extending mutations C-terminal to GFPGER at positions G508S and G511S GARGERGFPGER**G**VQ**G**PP as well as a mutation three triplets C-terminal to the insertion resulted in normal integrin binding and cell adhesion, similar to that of the VCL-Int control. This represents a very sharp cutoff C-terminal to the GFPGER sequence. The Arg-507 of collagen interacts with the I domain in the crystal structure, but the MD simulation shows that the salt bridge between the collagen Arg-507 and Asp-219 of the integrin is unaffected in the integrin-G508S complex.

There was a dramatic asymmetry in directionality for the footprint of binding disruption by a Gly to Ser mutation (Fig. 4*B*). Extending mutations to the two triplets N-terminal to GFPGER (G499S and G496S) **G**AR**G**ERGFPGERGVQGPP resulted in very low binding for the integrin I domain and diminished cell adhesion. To define the N-terminal boundary of this collagen integrin interaction footprint, more recombinant proteins were generated to extend Gly to Ser mutations further N-terminal to the human α1(I) six-triplet insertion, now going into the bacterial collagen sequence. The results indicated two more constructs of low binding, G(−3)S and G(−6)S, and one of intermediate binding G(−9)S, before reaching the mutations at sites G(−12)S and G(−15)S, which showed normal integrin binding and cell adhesion.

The effects of the mutations in the two triplets (G499S and G496S) N-terminal to GFPGER may be related to an inherent directionality of triple helix disruption. Molecular dynamics simulations on triple helical peptides with the sequences and mutations studied here indicated that each Gly to Ser replacement usually disrupts one to two direct NH···CO hydrogen bonds between each pair of chains within the triple helix and that the disruption extends preferentially to the C-terminal side of the Ser replacement. These computational results are consistent with residue-specific hydrogen exchange and amide chemical shift temperature dependence NMR studies carried out on a model triple helical peptide with a Gly to Ser replacement that showed a greater disruption of hydrogen bonding for the Gly in the triplet C-terminal to the Ser compared with the Gly in the triplet N-terminal to the Ser mutation site (8). An increased perturbation C-terminal to an OI mutation was also supported by a machine-learning analysis and related peptide studies (39).

**FIGURE 8.**
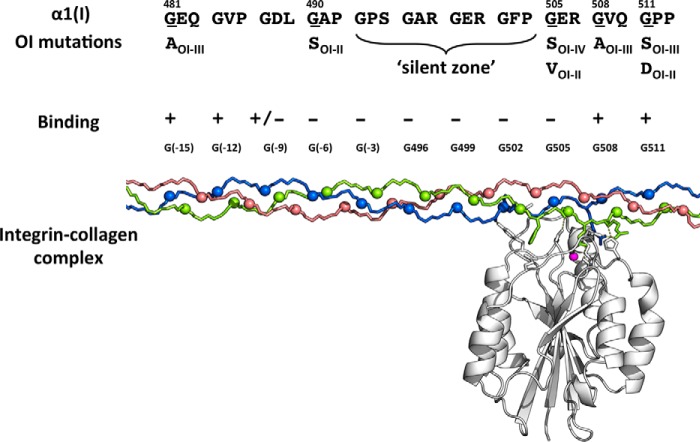
*Top*, known OI mutations in the integrin binding region as reported in the OI database (5, 6). The residue replacing Gly is shown below together with the Sillence classification of the phenotypic severity of the OI (*OI II*, perinatal lethal; *OI III*, severe; *OI IV*, moderate; *OI I*, mild) (7). *Middle*, the experimental effect of Gly to Ser mutations on integrin binding as reported here is indicated with + for normal binding and − for weak or no binding. *Bottom*, the molecular model of collagen-integrin binding along the triple helix shows the distance of N-terminal mutations from the well defined binding site.

Molecular dynamics simulations cannot explain how mutations three to five triplets N-terminal to the GFPGER (2.6–4.3 nm away given 0.29-nm rise/residue in the triple helix; Fig. 8) can affect binding. Hydrogen bond disruption extends from one to at most three tripeptides C-terminal to the mutation. It is possible that this region N-terminal to the GFPGER sequence plays a role in initial integrin recognition or relates to interactions with integrin dimers (40). Long distance effects of OI mutations have been reported previously by Lightfoot *et al.* (41, 42) where a register shift has been suggested to lead to a decreased N propeptide cleavage hundreds of residues away from the mutation site.

This is the first reported study on the effect of Gly missense mutations on a well defined biological interaction. The results show the expected disruption when the mutation is at the known interaction site as well as an unexpected disruption for mutations N-terminal to the known binding region. These results can be compared with mutations seen in the extensive database of known OI mutations within the human α1(I) collagen chain (771 mutations at 211 unique Gly sites). A non-lethal Gly to Ser missense mutation was reported at Gly-505 (GFP**G**ER) as well as mutations within Gly residues of the four triplets C-terminal to that site (Fig. 8). There are no reported mutations at Gly-502 (**G**FP) or the three triplets N-terminal to that site. It has been suggested that such a “silent zone,” containing four contiguous triplets with no reported mutations, could represent mutations that are non-viable, perhaps due to interference with ligand binding (7, 18). Such a stretch of four triplets with no known OI mutations is rare but could occur by chance. As more mutations are reported, it becomes less likely that a significant Gly-Xaa-Yaa stretch without mutations is due to chance. Our results indicate that mutations N-terminal to GFOGER do disrupt integrin binding, and such disrupted binding could create a silent zone. One other integrin binding region also seems to have such a silent zone, residues 811–830.

The recombinant bacterial collagen system allows easy modification of sequence and high protein yields to generate mutant collagens for binding studies. However, it is important to note that recombinant bacterial collagens are all homotrimers, whereas OI collagens are a mixture of heterotrimers containing zero, one, or two mutant α1(I) chains. Preliminary molecular dynamics simulation studies suggest that mutations in all three chains of the triple helix do not necessarily cause more distortion than when mutations are present in only one or two chains.^6^ The consequence of a Gly substitution on integrin binding is defined here using solid-state binding assays and cell adhesion. In OI cases, some mutant collagens may be degraded by the endoplasmic reticulum-associated protein degradation pathway or by autophagy. Only collagens that are secreted from the cell would participate in fibril formation, and fibrils containing a mixture of normal and mutant molecules would interact with cells. Despite the increased levels of complexity found in human OI mutations, the recombinant system provides focused information on the effects of collagen Gly missense mutations on integrin binding and gives insights into molecular mechanisms of collagen pathology.

## Experimental Procedures

### Design and Cloning of Constructs

The DNA sequence of bacterial collagen Scl2.28 was previously codon-optimized and inserted into the pCold-III vector (Takara Bio Inc., Japan) (43). Oligonucleotides encoding the sequence GARGERGFPGERGVQGPP from the human collagen α1 chain were designed with XmaI and ApaI sticky ends and synthesized (Thermo Fisher Scientific, Waltham, MA). Annealed dsDNA was cloned into pCold vector containing the Scl2.28 sequence that was previously digested by XmaI and ApaI. All enzymes were purchased from New England Biolabs (Ipswich, MA). Gly to Ser single base mutations were introduced using the QuikChange II kit (Agilent Technologies, Santa Clara, CA). Primers containing single base changes to convert the desired Gly codon, GGC, to a serine codon, AGC, were designed and synthesized (Thermo Fisher Scientific). Mutated plasmids were transformed into XL-1 Blue competent cells. DNA sequencing to confirm fidelity was carried out at Genewiz (South Plainfield, NJ).

### Expression and Purification of the Proteins

All constructs were expressed in the *E. coli* BL21 strain as described previously (43). The starting culture of 15 ml with 100 mg/liter ampicillin was grown overnight at 37 °C and used to inoculate 1 liter of LB-ampicillin medium in a flask. When *A* reached 0.8 at 600 nm, protein expression was performed by adding 1 mm isopropyl 1-thio-β-d-galactopyranoside and lowering the temperature to 20 °C. Cells were harvested by centrifugation after overnight expression. Cell pellets were resuspended with binding buffer (20 mm sodium phosphate, 500 mm NaCl, 10 mm imidazole, pH 7.4) and frozen at −20 °C overnight. Thawed cells were lysed by sonication and centrifuged at 4 °C to remove the cellular debris. Crude purification was done by immobilized metal affinity chromatography. The supernatant was loaded onto a HisTrap HP 5-ml column (GE Healthcare) using an fast performance liquid chromatography (FPLC) system (ÄKTA pure, GE Healthcare). The cellular extract was applied onto the column and purified with elution buffer (20 mm sodium phosphate, 500 mm NaCl, 500 mm imidazole, pH 7.4) after column equilibration. Elution fractions containing the protein were combined and dialyzed against 1× phosphate buffer saline (10× PBS, pH 7.4; Fisher Scientific). For further purification, the dialyzed samples were loaded onto a Hi Load 16/60 Superdex 200 prep grade size exclusion chromatography column (GE Healthcare) and eluted with 1.5 bed volumes of 1× phosphate buffer. All fractions containing the protein were collected and verified for purity level with SDS-PAGE (NuPAGE Bis-Tris 4–12%, Thermo Fisher Scientific) after denaturation at 75 °C in the presence of lithium dodecyl sulfate buffer (4×, Thermo Fisher Scientific). Electrophoresis conditions were 180 V, 120 mA for 40 min. Gels were stained with SimplyBlue stain for the visualization of protein bands (Thermo Fisher Scientific). Molecular weight determination was made by matrix-assisted laser desorption/ionization time of flight (MALDI-TOF) mass spectra (Bruker Corp., Billerica, MA). Concentrations of the protein samples were determined using UV-visible spectra at 280 nm (Spectrophotometer Model 14 UV-Vis, Aviv Biomedical Inc., Lakewood, NJ).

Proteins were denoted as VCL for the original Scl2.28 containing the V trimerization domain and the (Gly-Xaa-Yaa)_79_ CL domain from *S. pyogenes* and VCL-Int for the VCL protein containing the six-triplet human type I collagen α1(I) residue ^496^GARGERGFPGERGVQGPP^513^ insertion. The VCL-Int constructs with Gly to Ser mutations were denoted by the number of the residue in the human α1(I) chain that is mutated. For example, the construct with the mutation at the Gly-505 residue (GFP**G**ER) was denoted as G505S. Residue numbers are taken from UniProt entry P02452 for human α1(I) with numbering starting from the beginning of the triple helix region. After creating mutations in all six Gly residues within the human α1(I) insertion, it was necessary to go further N-terminal within the bacterial collagen sequence to find a mutation that did not affect integrin binding. These more N-terminal mutations fall within the bacterial collagen sequence and are denoted by their position relative to the human sequence insertion (*e.g.* using G(−3), G(−6), and G(−9) to denote the positions G(−9)*XY*G(−6)*XY*G(−3)*XY*GARGERGFPGERGVQGPP).

### Structural Analysis of the Expressed Proteins

#### 

##### CD Analysis

Wavelength scans were collected on an AVIV Model 420 CD spectrometer (AVIV Biomedical Inc.) between 190 and 260 nm with 0.5-nm increments, 4-s averaging time, and 1.0-nm band width at 5 °C as described previously (44). Samples were prepared with 0.5 mg/ml concentration in 1× PBS, and 1-mm quartz cuvettes were used. Temperature scans were collected at 220 nm from 5 to 70 °C with 10-s averaging time and 1.5-nm band width. The heating rate was an average of 0.1 °C/min.

##### Differential Scanning Calorimetry Analysis

Differential scanning calorimetry profiles were obtained on a NANO DSC II Model 6100 (TA Instruments, New Castle, DE). Each sample was referenced with its own dialysis buffer with 1× PBS. Measurements were taken from 5 to 70 °C with a heating rate of 1 °C/min.

##### Trypsin Cleavage Assay

All proteins (*c* = 0.1 mg/ml in 1× PBS) were trypsinized with 50 μl of trypsin solution (10 μg/ml; 0.4 μm in 1× PBS) by incubating at 20 °C for 2, 5, and 15 min. Trypsinization was stopped by adding 1 mm phenylmethylsulfonyl fluoride (PMSF). The trypsin digestion profile of the constructs was analyzed by SDS-PAGE. Band integrated densities were analyzed by ImageJ.

### Functional Analysis of the Constructs

#### 

##### Solid-state Binding Assay

A previously published solid-state binding protocol to assess integrin I domain binding to triple helical peptides was followed (21, 46). Different concentrations of the recombinant collagens were tested to find the optimal concentration for coating and binding to the I domain, and that concentration (0.02 mg/ml) was used in all assays. An aliquot of 100 μl of purified recombinant collagen (0.02 mg/ml) was immobilized onto high binding 96-well plates (R&D Systems, Minneapolis, MN) for 1 h at room temperature. Blocking (50 mg/ml BSA in 1× PBS) and washing (1 mg/ml BSA in 1× PBS) steps were repeated. Aliquots of 100 μl of recombinant integrin α2 I domain (20g/ml in 1× PBS with 2 mm MgCl_2_) were added onto the coated wells and incubated for 1 h at room temperature. Subsequently, wells were washed with 200 μl of washing buffer (2 mm MgCl_2_ added), and 100 μl of anti-GST HRP antibody (1:10,000 dilution in washing buffer with 2 mm MgCl_2_) was added onto each well and incubated for 1 h at room temperature (Sigma-Aldrich). 3,3′,5,5′-Tetramethylbenzidine solution (Invitrogen) was used for colorimetric detection. The *A*_450 nm_ was recorded with a Spectra Max M2 plate reader (Molecular Devices, Sunnyvale, CA). Denatured proteins were used to verify the integrin binding selectivity for native triple helix. All proteins (20 μg/ml) were denatured at 75 °C for 30 min and immobilized onto the well plates immediately. Native type I collagen from rat tail (Sigma-Aldrich) was used as the positive control for all the binding assays. The procedure was repeated without the addition of 2 mm MgCl_2_ to the washing and adhesion steps as a negative control for verifying the mode of binding. All assays were performed in triplicate.

For dose-response assays, serial concentrations of I domain at 90, 20, 4, 2, 1.2, 0.9, and 0.18 μg/ml were used, corresponding to ∼1.8, 0.4, 0.08, 0.04, 0.024, 0.009, and 0.0018 μm I domain in molar concentration.

##### Mammalian Cell Culture

Human fibrosarcoma HT1080 cells (ATCC, Manassas, VA) were used for cell adhesion assays (performed in triplicate). High binding 96-well plates (R&D Systems) were coated with 60 μg/ml recombinant proteins for 1 h at room temperature. Blocking was performed by coating the wells with 200 μl BSA solution (50 mg/ml in 1× PBS) for 1 h at room temperature. Wells were washed with 200 μl of washing buffer (1 mg/ml BSA in 1× PBS) four times. 100 μl of HT1080 cells (2 × 10^4^cells/ml) in DMEM (Invitrogen) were seeded onto wells and incubated for 1 h at 37 °C (supplemented with 10% FBS and 2 mm MgCl_2_ with 5% CO_2_ supply). Non-bound cells were washed with 200 μl of DMEM (no FBS; 2 mm MgCl_2_ added) four times. For cell imaging, 100 μl of 2 μl of calcein-AM solution (Sigma-Aldrich) was added onto each well and incubated at 37 °C for 30 min. After washing away excess calcein with DMEM, fluorescence images of cells were taken with 490-nm excitation and 515-nm emission filters on a Leica DFC340 FX camera. In addition to live imaging, crystal violet staining was used to quantitate cell adhesion. Cells were fixed with 90% ethanol solution for 10 min. 0.1% crystal violet solution (Sigma-Aldrich) was added to each well and incubated for 30 min at room temperature. Solubilization of the dye was facilitated by adding 0.1% Triton X-100, and *A*_590 nm_ was recorded with an absorbance plate reader (Spectra Max M2).

##### Molecular Dynamics Simulations of Triple Helical Collagen Peptides

To investigate how Gly to Ser substitutions within and near the human α1(I) insertion affect the triple helical structure, model triple helical collagen peptides containing the wild-type (WT) collagen sequence and six mutants were simulated in this study, GPOGPOGPOGPAGPQGPRGEQGPQ**G**AR**G**ER**G**FP**G**ER**G**VQ**G**PPGLPGKDGEAGPOGPOGPO with Gly to Ser mutants at each of the underlined sites. The initial structure for the WT triple helical collagen was built using the Triple-Helical Collagen Building Script (THeBuScr) (47). The initial structure for the mutants was generated by replacing Gly at the corresponding position with Ser residues for chains A, B, and C using the UCSF Chimera package (48). The N and C termini for all the collagen models were capped with the acetyl group and NH_2_ group, respectively. Within the triple helix, the trailing chain is designated as chain A, the middle chain is designated as chain B, and the leading chain is designated as chain C following the notation of Emsley *et al*. (23).

All MD simulations in this study were performed using the GROMACS 4.6.7 suite (49) with the GROMOS 54a7 force field (50) and SPC water model (51). For each collagen triple helix, the starting structure was first energy-minimized and then solvated in a rectangular periodic box of water. The dimension of the box was chosen such that the minimum distance between any atom of the collagen and the box walls is 1.5 nm. The solvated system was further energy-minimized. With all heavy atoms of the collagen harmonically restrained, each system was heated from 5 K to the target temperature (300 K) within 20 ps and equilibrated for an additional 30 ps. Before production simulation, an additional 500-ps equilibration with only the collagen backbone restrained was also performed.

The 100-ns production runs were performed in the isobaric-isothermal (NPT) ensemble at the target temperature of 300 K and a pressure of 1.0 bar. The first and last Cα carbons of each chain were restrained with a force constant of 10 kJ/mol/nm^2^. All bonds were constrained with the LINCS algorithm (52). The non-bonded interactions were truncated at 0.8 nm. Long range electrostatic interactions beyond the cutoff distance were calculated using the particle mesh Ewald summation method (53). A long range analytic dispersion correction was applied to both the energy and pressure to account for the truncation of Lennard-Jones interactions (45).

##### Molecular Dynamics Simulations of Integrin-Collagen Peptide Complex

To provide detailed insight into the consequences of Gly to Ser substitutions on integrin-collagen binding, the complex of integrin α2β1 I domain bound to the WT or G502S/G505S triple helical collagen peptide was also simulated. The initial coordinates for the integrin I domain and triple helical GFPGER sequence were taken from the x-ray structure (Protein Data Bank code 1DZI) (23). The coordinates for the atoms of the WT collagen peptide not present in the x-ray structure were generated by aligning the THeBuScr built triple helix to the x-ray structure based on the Cα positions of the GFPGER sequence. The prepared complex structure was then subjected to 100 steps of energy minimization in vacuum with the integrin and GFPGER heavy atoms restrained to their initial positions. The initial structure for integrin binding to the G502S/G505S collagen peptide was built by substituting Ser residues for Gly at positions 502/505. In the MD simulations, the Co^2+^ ion was replaced by a Mg^2+^ ion. All water molecules found in the x-ray structure were retained.

The integrin-collagen complex structure prepared above was solvated in a rectangular water box of size 200 × 80 × 80 Å^3^. One chloride ion was also added to neutralize the net charge of the solvated system. With all heavy atoms of the integrin and collagen restrained, the system was further energy-minimized followed by 20-ps heating from 5 to 300 K and 30-ps equilibration. Before the production run, an additional 100-ps equilibration with only the collagen backbone restrained was also performed. The 50-ns production runs were performed using the same protocol as described in the previous triple helical collagen peptide simulation section.

## Author Contributions

S. Y., H. Y., Y.-S. L., and B. B. designed the study and wrote the paper. S. Y., H. Y., S. H., D. L. K., R. W. F., Y.-S. L., and B. B. analyzed and critically evaluated the results. S. Y. performed experiments with recombinant bacterial collagen and HT1080 cell adhesion assays. H. Y. performed the MD simulations. B. A. aided in cloning experiments. S. H. and R. W. F. provided the recombinant I domain and expertise on integrin binding assays and cell adhesion. All authors read and approved the final version of the manuscript.

## Footnotes

5 Molar concentrations listed in the paper were single chain concentrations calculated based on the single chain molecular mass of I domain at ∼50 kDa, VCL-Int at ∼34 kDa, and animal collagen at ∼95 kDa. I domain constructs are dimers due to the GST tag, and collagen is a trimer.

6 A. Mekkat, H. Yu, and Y.-S. Lin, personal communication.

## Footnotes

4 The abbreviations used are:

Hyp: hydroxyproline (O, one-letter amino acid notation)
OI: osteogenesis imperfecta
MIDAS: metal ion-dependent adhesion site
MD: molecular dynamics
Bis-Tris: 2-[bis(2-hydroxyethyl)amino]-2-(hydroxymethyl)propane-1,3-diol.
